# The Source and Distribution of Tetracycline Antibiotics in China: A Review

**DOI:** 10.3390/toxics11030214

**Published:** 2023-02-24

**Authors:** Donghao Chang, Yizhi Mao, Wei Qiu, Yunshu Wu, Baiyan Cai

**Affiliations:** Engineering Research Center of Agricultural Microbiology Technology, Ministry of Education & Heilongjiang Provincial Key Laboratory of Ecological Restoration and Resource Utilization for Cold Region & Key Laboratory of Molecular Biology, College of Heilongjiang Province & School of Life Sciences, Heilongjiang University, Harbin 150080, China

**Keywords:** tetracycline antibiotics, different media, distribution characteristics, pollution status, residues

## Abstract

In recent years, antibiotics have been listed as a new class of environmental pollutants. Tetracycline antibiotics (TCs) used in human medical treatment, animal husbandry and agricultural production are the most widely used antibiotics. Due to their wide range of activities and low cost, their annual consumption is increasing. TCs cannot be completely metabolized by humans and animals. They can be abused or overused, causing the continuous accumulation of TCs in the ecological environment and potential negative effects on non-target organisms. These TCs may spread into the food chain and pose a serious threat to human health and the ecology. Based on the Chinese environment, the residues of TCs in feces, sewage, sludge, soil and water were comprehensively summarized, as well as the potential transmission capacity of air. This paper collected the concentrations of TCs in different media in the Chinese environment, contributing to the collection of a TC pollutant database in China, and facilitating the monitoring and treatment of pollutants in the future.

## 1. Introduction

Antibiotics are secondary metabolites produced by microorganisms (such as actinomycetes, bacteria and fungi) or higher plants with antipathogenic properties [1,2]. Currently, antibiotics are obtained by chemical synthesis or by chemical modification of natural compounds [3]. Antibiotics have good therapeutic and preventive effects on bacterial diseases and are mainly used in the fields of human medicine (surgery, chemotherapy or organ transplantation and treatment of infectious diseases) [4], breeding industry (animal husbandry, beekeeping and fish) and agriculture (vegetables and fruit trees) [5]. It is estimated that 75% of patients with seasonal influenza receive an antibiotic prescription [6]. It is estimated that by 2030, the total amount of antibiotics used in China’s livestock industry will account for 30% of global production [7]. The global consumption of antibiotics in agriculture has increased by 67% [8]. Another source of antibiotics is aquaculture, where China accounts for approximately 71% of global aquaculture production [9]. It has been stated that 70–80% of the antibiotics used in fish farms end up in the environment [10]. Human overreliance on antibiotics and the excessive pursuit of economic interests have led to the inappropriate long-term use of high-dose or high-frequency antibiotics, resulting in the continuous release of antibiotics into the environment. The addition of antibiotics to feed to promote animal growth has been banned in the EU. However, antibiotics are still widely used in animal husbandry in developing countries (China and India) [11,12]. China is the world’s largest producer and consumer of antibiotics, with an annual output of approximately 210,000 t, of which 48% were applied in agriculture, 42% in medicine and the remaining 10% were exported [13,14]. The increased consumption of antibiotics by humans and animals has led to growing concerns about adverse ecological effects due to their ”pseudo-persistence” in the environment [15]. The stability of trace pollutants is not high or easy to degrade, but because the pollution source will continue to input into the environment, it will maintain a certain concentration in the environment, known as “pseudo-persistent”. Animal manure, soil, sediment, sludge, wastewater (domestic sewage, industrial wastewater and medical wastewater), groundwater, tap water and surface water (springs, lakes, streams, rivers and oceans) are reported to be contaminated with antibiotics [16,17,18].

**Figure 1:**
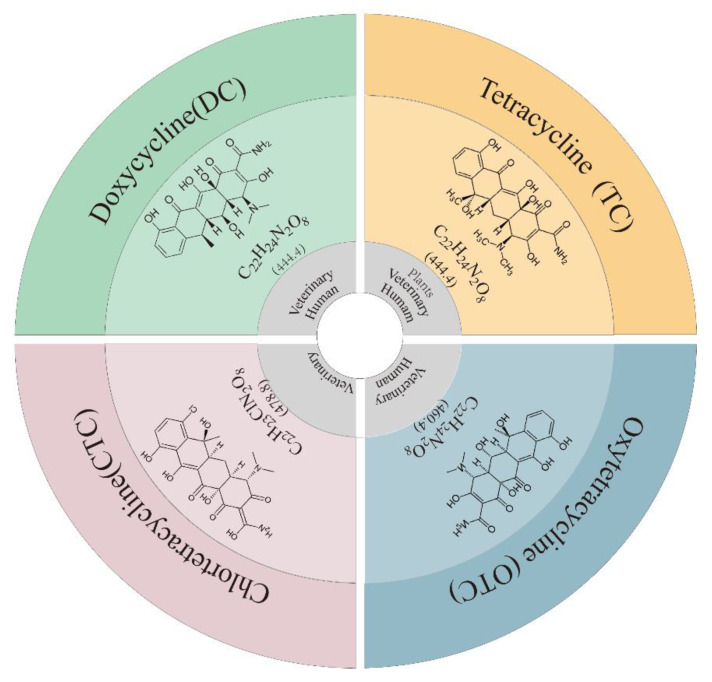
Physical and chemical properties of TCs. Outermost circle is name and abbreviation; middle is structural formula, molecular formula and molecular weight; and inner circle is main use.

The first mention of TCs in the scientific literature dates back to 1948, and they started to be commercialized with good clinical results before long [19]. TCs are a family of broad-spectrum antibiotics that inhibit protein synthesis by preventing aminoacyl-tRNA from attaching to the ribosome, thereby achieving bacteriostatic effects [20]. TCs are widely used in aquaculture and livestock because they are inexpensive, simple to use and have relatively minor side effects [21]. TCs include natural, semisynthetic and synthetic compounds. Twenty compounds have been introduced in the market as antibiotics. The most widely used antibiotics in veterinary medicine are tetracycline (TC), oxytetracycline (OTC), chlortetracycline (CTC) and doxycycline (DC), which are classified as priority medications in both aqueous and solid phases [16]. The chemical structures of TCs are strikingly similar to those of other TCs (Figure 1) and are derived from a core of hydronaphthacene that shares four fused rings [22]. Thousands of tons of TCs are thought to be consumed annually by both people and animals in the European Union. The excretions of up to 75% of ingested TCs are in their active forms, and large volumes of TCs enter the environment [23].

With the rapid development of the global economy, people tend to ignore the importance of the ecology, causing great pollution to the environment. The unprecedented use of TCs in treating and preventing diseases in humans and in aquaculture and agriculture has resulted in the continuous influx and accumulation of TCs. They can disrupt the safety and diversity of organisms in the environment and pose a serious threat to human security. This review summarizes the distribution characteristics of TCs in various environmental media and assesses the residual situation of their environment. In addition, this paper focuses on the exposure and danger levels of TCs in China’s ecological environment and provides valuable reference information for protecting the ecological environment.

## 2. Occurrence of TCs in the Environment

Using the Web of Science (https://www.webofscience.com, accessed on 16 May 2022) and China academic journal network publishing database (https://www.cnki.net, accessed on 16 May 2022) for information retrieval, the two databases almost covered all the scientific literature in both Chinese and English. All search terms within a string were checked for a “title, abstract, and keyword”. The search algorithms were “tetracycline antibiotics”, “environment” (“soil”, “water” or “air”) and “China”. Considering the adequacy of the amount of data and the consistency of data accuracy, the relevant literature in the past ten years (2012–2022) was selected. According to the results of the investigation in the past 10 years, the environment in all parts of the country has been polluted by antibiotics to varying degrees. Although the use of antibiotics has a history of nearly a hundred years, the research on its environmental risk is still under exploration.

**Figure 2:**
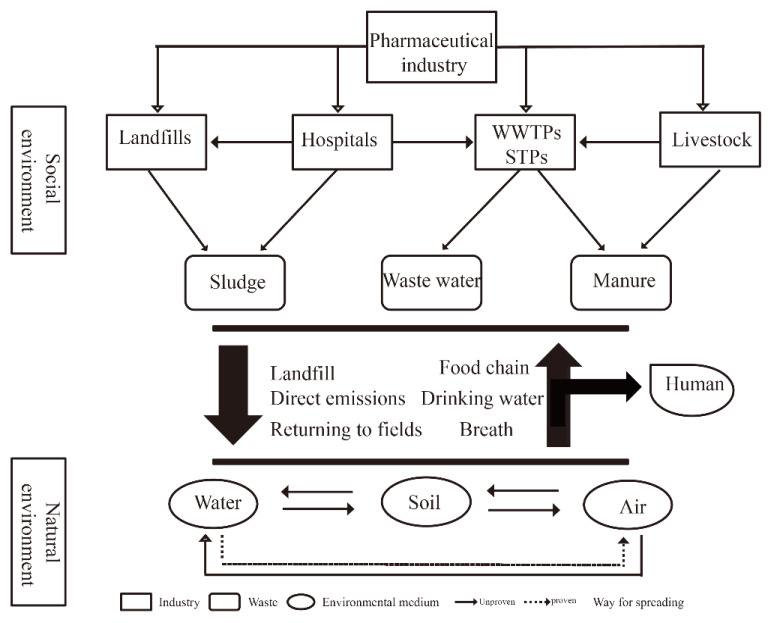
Main transport routes of TCs in the environment.

There are two main ways in which TCs enter the environment (Figure 2). One source is the excreta of humans, livestock, fish and other animals. Since TCs are difficult to completely absorb and metabolize in animals, most of them remain in the excreta as original drugs or intermediate metabolites. With concentration ranges of 1.16–2.09, 1.2–3.76, 0.94–41.2 and 5.98–23.79 ng/mL, respectively, TC, OTC, CTC and DC were found in fresh human urine samples [24]. The other source is TCs that are contained in the wastewater and waste generated by pharmaceutical factories, hospitals and sewage treatment plants during production and use. At pharmaceutical wastewater treatment plants (WWTPs), large amounts of TCs were discharged through the effluent (up to 32.0 ± 6.0 mg/L) and dewatered sludge (up to 5481.1 ± 123.0 mg/kg) [25]. The concentrations of TC detected in hospital sewage were 330–1240 ng/L [26]. The concentration of TC in the sewage of Beijing Hospital was 20.81–76.33 ng/L [27]. A large number of unused and expired medicines were directly used as domestic waste, and more than 90% of the waste was still directly landfilled without pretreatment [28]. The concentrations of TC and OTC detected in landfills were 63.8 ± 37.7 μg/kg and 100.9 ± 141.81 μg/kg, respectively [28,29]. Through methods such as direct discharge, sewage treatment facilities and landfills, TCs may reach the environment directly or indirectly. TCs are emerging environmental pollutants. When they enter water or soil, they cause great harm to the ecosystem and adversely affect human health and safety.

Currently, TCs are manufactured by pharmaceutical industries and supplied to various industries, and finished medicines are supplied to the pharmaceutical system (antibiotics for humans) or farms (antibiotics for veterinary use). Sewage generated from the pharmaceutical process enters the sewage plant for treatment, and some solid waste (leftovers or expired medicines) enters garbage plants for landfill treatment. A sewage treatment plant will treat wastewater and domestic sewage from hospitals and breeding plants. However, the plant cannot absolutely eliminate the TCs, and the sludge produced contains high concentrations of TCs. This is used as organic fertilizer and animal husbandry manure for returning to the field and is directly discharged into rivers for the fish farming industry. In the natural environment, TC pollution is mainly due to waste, sewage and organic fertilizers generated by the social environment.

## 3. Distribution of TC Pollution

### 3.1. Contamination Status of TCs in Animal Manure

The rapid development of livestock and poultry farming has become an important industry in China, and TCs have become the most widely used veterinary antibiotics in China [18]. According to reports, TCs are frequently added to the food given to animals of all ages in order to boost growth and ward off sickness [11,12,30]. Three antibiotics (TC, OTC and CTC) were found in feed from farms in Guangxi Province, at maximum concentrations up to 71,800 ± 8860 μg/kg, 4160 ± 658 μg/kg and 12.1 ± 0.72 μg/kg, respectively [30]. They were excreted in the urine and feces in their original active form. The excretion rates of TC, OTC and CTC after animal medication were at levels of 72%, 21% and 65–75%, respectively, and TC excretion may also vary with animal type, dosage, feed brand and growth stage [8,31,32]. 

The output of manure around the farm is large, and this manure is directly applied to the surrounding soil without comprehensive treatment, resulting in the pollution of antibiotics in the soil. In China, antibiotics are added to feed in excess of 8000 t annually, while the annual maximum production of livestock waste is predicted to reach 3.19 billion tons [33,34]. In China, just 25% of the total amount of fertilizer used is made from commercial organic fertilizer, which accounts for less than 40% of the animal dung produced [35]. Several studies have investigated TC, OTC and CTC, which have half-lives of 4.5–105 d, 3.2–30 d and 1–86.8 d in sludge or fecal composting, respectively [36]. TCs have previously been detected in feces from various animals at levels ranging from the low μg/kg range up to the mg/kg range [37,38]. The residual amount of TCs found in animal waste is listed in (Table 1). It can reflect the use of tetracycline antibiotics and the concentration that may enter the environment. The residue of TCs in feces from different regions of China is of the order of mg/kg. Animal species have an impact on the amount of TC residues in feces, but other elements including processing technique and storage period also have a role.

Residue levels of TCs are in the order pig manure > poultry manure > cow manure and are generally higher in manure from industrial farms compared to manure from farm households. Northeast China is composed of Heilongjiang, Jilin and Liaoning provinces. CTC is the most commonly used, while OTC is the most used; the utilization of TC in pig feeding farms was more common than in chicken and cow feedlots [31]. The TCs are less persistent in pig manure and are generally moderately to very persistent in most cow and chicken manures. The most plausible explanation is that a considerable fraction of the TCs bond to solid particles instantly, and the strong binding delays biodegradation [36,38]. It is challenging to determine the concentrations of TC residues in real-world situations because TC migration from manure to soil is a complicated process. This takes into account the intermediate processes during TC migration such as dilution, degradation, plant absorption and leaching [39]. Liu et al. repeated applications of biogas slurry that did not generate an excessive buildup of antibiotic residues in soil, presumably because the applications of biogas slurry were made over a relatively short period of time (1–5 years) [40]. Lu et al. studied the effects of long-term (8–18 years) biogas slurry application on soil OTC and CTC residue concentrations and ARG abundance [41]. Kyselková et al. showed no direct association between untreated and OTC-treated cow excreta and soil resistance genes [42]. This shows that the effects of TCs in soil are influenced not only by soil properties but also by animal species and gut microbiota [42,43].

**Table 1:** TCs’ residual content in animal feces.

Area	Animal	TC	OTC	CTC	References
Three northeastern provinces ^a^ (mg/kg)	Dairy cow	0.43–2.69	0.21–10.37	0.61–1.94	[31]
	Chicken	0.54–4.57	0.96–13.39	0.57–3.11	
	Pig	0.32–30.55	0.73–56.81	0.68–22.34	
Shenyang (mg/kg)	Pig and Chicken	0.06–56.95	0.57–47.25	1.24–143.97	[44]
Shanghai (mg/kg)	Cattle	12.01	21.36	/	[33]
	Chicken	10.31	21.96	/	
	Pig	12.27	18.70	/	
Zhejiang (μg/kg)	Cattle	1704	1120	108–778	[45]
	Chicken	424–9376	5065	981–5628	
	Pig	589–4145	524–16,280	340–15,872	
Eight provinces of China ^b^ (μg/kg)	Cattle	17.30–2495	130–1940	15.10–65.5	[46]
	Chicken	21.40–8675	19.0–416,750	13.90–129.5	
	Pig	15.90–30,941	21.50–43,429	20.6–215,346	
Jiangsu (μg/kg)	Cow	3.41	6.20	0.85	[47]
	Swine	187.70	108.70	84.60	
	Chicken	3.61	290.50	125.90	
Eight provinces of China ^c^ (mg/kg)	Cow	/	0.32–59.59	0.24–27.59	[48]
	Pig	/	0.15–59.06	0.16–21.06	
	Chicken	/	0.27–10.56	0.16–17.68	

Concentration range of each antibiotic in different regions (minimum–maximum) or (maximum) concentrations. ^a^: Three northeastern provinces: Heilongjiang, Jilin and Liaoning provinces. ^b^: Eight provinces of China: Chengdu, Kunming, Xuzhou, Nanjing, Shanghai, Shouguang, Wuhu and Hangzhou. ^c^: Eight provinces of China: Guangxi, Hubei, Hunan, Jiangsu, Jiangxi, Shandong, Shanghai and Zhejiang.

### 3.2. Pollution Status of TCs in WWTPs

WWTPs are the last line of defense against harmful substances in domestic and industrial wastewater entering the environment. The removal efficiency of antibiotics depends on the physicochemical characteristics [48]. The treatment processes include physical (membrane filtration), biological (membrane bioreactors) and chemical techniques (chlorination, advanced oxidation) [49,50,51,52]. In sewage treatment plants, antibiotics are removed by hydrolysis, filtration, biodegradation and adsorption into sludge. WWTPs were not originally designed to treat pharmaceutical contaminants; with the development of existing technologies, many organic pollutants can be removed, but the ability of WWTPs to remove antibiotics is limited [25]. The presence of TCs and metabolites in WWTPs varies according to the source and level of antibiotic consumption. The effect of TCs’ removal is different in different WWTPs: there are still TCs in the effluent, and most of them are stored in sludge (Table 2). The residues of TCs in the effluents discharged from WWTPs in different regions of China are very low, and the overall ecological risk of the effluents is low, with all average values less than 100 µg/kg and most of them less than 10 µg/kg. Most TCs are transferred to sludge. There are high maximum residual TCs concentrations (>1 mg/kg) in sludge from Xiamen and Shenyang and low maximum residual TCs concentrations (<100 μg/kg) in sludge from Guangdong and low ecological risk.

Sewage sludge is an inevitable waste produced by urban sewage treatment facilities. The treated wastewater containing antibiotics in WWTPs is used for irrigation or discharged into natural water bodies, resulting in a concentrated source of typical antibiotics in rivers. With potential agricultural nutritional value, sludge is a complex heterogeneous mixture of microorganisms and undigested organics such as paper, plant leftovers, oils, fecal waste and inorganic components [53]. In total, 6.25 million tons of dry solids were created in 2013 in China, where the total volume of sludge increased by an average annual growth rate of 13%. More than 80% of the sludge was dumped, with a tiny fraction being disposed of through sanitary landfills [54]. Sludge has been utilized widely as a fertilizer for the land up to now [55]. There is a high concentration of TCs in the sludge because a large number of drugs are adsorbed on the surface of the solid particles in the sludge. Through an electrostatic interaction with positively charged cations, adsorption occurs, or complex and cation exchange reactions with metal ions occur in the sludge. Approximately 70% of undegraded antibiotics are transferred to sludge, also with clays, natural organics, metal oxides via cation exchange, bridging hydrophobic partitioning and electron donor–acceptor interactions [56,57]. If the sludge is returned to the field, such a high concentration of TCs in the sludge will have a negative impact on the farmland soil environment, including affecting microbial diversity and respiration.

**Table 2:** Residual content of TCs in WWTPs.

Area		TC	OTC	CTC	DC	References
Guizhou	sewage (ng/L)	478.64–20.52	196.32–24.12	254.96–17.76	132.66–12.27	[58]
	sludge (μg/kg)	120.25	80.27	77.98	65.51	
Xiamen	sewage (ng/L)	179–18.00	293–22.70	24.30–4.43	/	[56]
	sludge (μg/kg)	4870	1710	1260	/	
Guangdong	sewage (ng/L)	39.20–4.85	57.70–3.50	4.85–0.57	3.88–0.17	[30]
	sludge (μg/kg)	15	20.60	4.16	3.31	
Eight provinces of China ^a^	sewage (ng/L)	110.10–25.80	626.90–64.50	39.40–3.80	23.70–4.50	[59]
	sludge (ng/kg)	1038.40	5115.90	276.60	112.60	
Guangzhou	sewage (ng/L)	36.30–3.60	87.8–6.79	36.4–10.60	25.1–7.41	[60]
	sludge (ng/kg)	269	437	57.20	29.10	
Beijing	sewage (ng/L)	490.30–10.69	16.50–6.32	9.25–nd	/	[27]
Shenyang	sludge (μg/kg)	297.12–2174.46	174.21–7369.67	197.39–3843.79	127.45–2104.27	[48]

Concentration range of each antibiotic in different regions (influent—effluent) or (maximum) concentrations. nd: not detected. ^a^: Chongqing, Qinghai, Shanghai, Jiangsu, Beijing, Shanxi, Dalian, Shandong.

### 3.3. Contamination Status of TCs in Soil

Antibiotic-contaminated manure is frequently used as a soil amendment or land fertilizer in China, Europe, the Americas and other regions of the world [61]. Manure is high in organic matter, nitrogen and phosphorus, which can enhance the physical and chemical properties of soil and provide agriculturally essential nutrients [62]. The majority of antibiotics used in medicated feeds is excreted in feces. Although antibiotics are partially degraded during composting or anaerobic fermentation, the large application amount and repeated application cause TCs in the form of the parent or an individual. Metabolites enter the soil and become one of the main ways to pollute the soil. River or lake water contaminated by TCs is often used for agricultural irrigation, which forms another important pathway [39].

Detectable concentrations of TCs in soil range from μg/kg to mg/kg (Table 3), and TCs have higher partition coefficients than other antibiotic classes and are more readily absorbed by soil particles, suggesting that these compounds are preferentially retained in soil [33,35]. More than half of the literature on soil TC pollution in China focuses on the application of livestock and poultry manure or vegetable bases near the farm. The concentrations of TC, OTC, CTC and DC in Chinese agricultural soils varied as 0–977 μg/kg, 0–8400 μg/kg, 0–5520 μg/kg and 0–871 μg/kg, respectively. TC pollution in Shenyang, North China is more serious than that in other areas. The ecological risk of TCs in the soils of Guangdong, Beijing and Zhejiang provinces is generally low, with all averages less than 100 μg/kg. The pollution degree of TCs is also different in different areas of Fujian province. The dispersion of antibiotics may be caused by various factors such as manure type, fertilization amount, fertilization method, planting density, soil properties, TC half-life or planting years [63]. In soil, 100 μg/kg is the threshold value for antibiotic ecotoxicity [64]. TCs are in the cationic form, which can have a strong adsorption effect on negatively charged soil components. During the physical adsorption process, chelates form with multivalent cations such as Ca^2+^, Cd^2+^, Mg^2+^ and Al^3+^ or form ternary complexes as a bridge between organic matter and TCs [65,66,67]. The process of forming chelates or complexes is reversible, reducing the acute toxicity of TCs and storing them in a bioavailable form, thereby prolonging the residence time of TCs in soil. The physical diffusion of nanopores and other parts in the interlayer and the formation of covalent bonds catalyzed by enzymes form non-extractable residues, making antibiotics impossible to extract from soil solutions [8].

The pH, texture, metal ions, clay content, organic matter, rainfall duration and pH of the rain all have a substantial impact on the adsorption and migration of antibiotics in the soil [68,69,70]. Long-term fertilizer application significantly increases TC residue, TCs were not detected in unfertilized soils, and multiple studies confirmed that antibiotics enter agricultural soil environments through fertilization [34,36]. Long-term exposure to antibiotics in agricultural soils after repeated fertilization increases the accumulation of antibiotics and accelerates their biodegradation, and their whereabouts and accumulation remain unclear [34,71]. The concentration of TCs in farmland soil is significantly different in summer and winter. The concentration of TCs in farmland soil is higher than that in orchards and forests. With increasing soil depth, the concentration of TCs decrease [68]. It is critical to look at the concentration and distribution of antibiotics in soil given the rise in TC detection in soil samples and their possible impact. Animal manure is applied frequently and for long periods of time, adding nutrients and increasing TC residues at the same time. Polyethylene, polypropylene and polyvinyl chloride all have the potential to adsorb TC under laboratory conditions and are significantly affected by pH and organic matter. TC has strong adsorption to humic acid, complexation and hydrogen-bonding interactions with humic acid [72].

Research shows that bacteria in feces may not be well adapted to the soil environment because bacterial communities in feces and soil are largely different. [73]. After adding TCs to the soil, the growth of the bacterial community was significantly inhibited, and this continued even after the incubation period of 42 d; however, the toxicity to bacteria was also weakened [74]. There are several causes for the resumption of bacterial growth. The bioavailability of these compounds to the bacterial community is decreased by the degradation and adsorption of TCs in the soil, which lower the selection pressure on bacteria. In addition, drug resistance or bacterial colonies form protective membranes [74,75]. TC also inhibits enzyme activity in the soil. It significantly affects the activity of catalase, dehydrogenase and urease at low and high OTC concentrations [76]. Different OTC levels had rather predictable impacts on soil dehydrogenase activity, with greater contents promoting it more in the early phases of incubation and lower contents inhibiting it. Meanwhile, the activity of soil neutral phosphatase was unaffected by OTC up to 30 mg/kg in soil [77]. The composition of the soil microbial community was reportedly affected by TC, which did not affect the fungal population but definitely stimulated actinomycete bacteria, antibiotic makers and other soil microflora [78].

**Table 3:** TCs’ residual content in soil.

Area	TC	OTC	CTC	DC	References
Fujian (μg/kg)	UQ	11.30–79.50	nd–58.80	/	[79]
	UQ–189.80	7.20–613.20	UQ–2668.90	/	
	UQ–45.40	8.3–23.10	UQ–864	/	
	UQ–58.10	44.7–219.10	nd–240	/	
Shanghai (mg/kg)	2.38	4.24	/	/	[33]
Zhejiang (μg/kg)	nd–40.46	nd–42.22.	nd–460.80	/	[80]
Shenyang (μg/kg)	29.51–976.17	17.62–1398.47	8.29–1590.16	11.05–870.45	[44]
Guangdong (μg/kg)	nd–74.40	nd–79.70	nd–104.60	/	[62]
Tianjin (μg/kg)	UQ–105	2.5–2683	nd–1079	/	[39]
Zhejiang (μg/kg)	(1.50)	6.72	1.70	16.40	[81]
Beijing (μg/kg)	nd	1.60 (0.80)	1.60 (0.60)	nd	[82]
Beijing (μg/kg)	2.60–5.40	13–42	3.90–14		[62]
Four provinces of China ^a^ (μg/kg)	60.44 (11.69)	415.00 (37.10)	222.00 (21.46)	94.10 (12.50)	[83]
Guangzhou (μg/kg)	0.16–25.66	0.04–31.85	0.29–161.50	0.87–184.80	[84]
Jiangsu (μg/kg)	1.3–249	1–8400	101.5	1.1–256	[85]
Jiangsu (μg/kg)	nd–763	nd–3511	nd–4723	nd–76.5	[86]
Four cities in China ^b^ (μg/kg)	nd–29.70	nd–40.27	23.84–344.74	/	[34]
	nd–18.92	nd–23.26	81.33–38.49	/	
	nd	nd	nd–31.35	/	
	nd–7.34	n.d–14.34	nd–107.86	/	

Concentration range of each antibiotic in different regions ”minimum–maximum” ”maximum” or (mea) concentrations. nd: not detected. UQ: unquantified concentration. ^a^: Hebei, Henan, Sichuan and Jiangsu. ^b^: Jiaxing, Changsha, Yingtan and Nanchang.

### 3.4. Pollution Status of TCs in Water Bodies

#### 3.4.1. TC Accumulation in Surface Water

Rivers around cities play a vital role in social and economic development. In addition to serving as a significant source of drinking water for both urban and rural inhabitants, they also provide water for aquaculture, ecological landscapes and agricultural irrigation. The temporal and spatial distributions of antibiotics in water sources in different regions of China are quite different. On the one hand, this difference is closely related to the local industrial structure; the method of antibiotic disposal in the pharmaceutical industry; the use of antibiotics in livestock, poultry and aquaculture; and the flood season of rivers. Sewage, surface runoff and direct discharge from animal husbandry resulted in high TC content in some surface water samples in China, and the residual content of TCs in surface water was collected (Table 4). The range of residual TC concentrations in the lake, river and sea in different regions of China varies widely, ranging from 0–2199.50 ng/L, 0–97,434 ng/L and 0–914.06 ng/L, respectively. The pollution of TCs in Honghu Lake in Hubei province is relatively serious, and the ecological risk of TCs in Honghu Lake is higher; all averages are more than 100 μg/kg. The TCs’ content of Poyang Lake, Chaobai River Hai River, Guangdong coastal areas and other watersheds varies irregularly with the change of sampling sites. TCs in surface water are significantly positively associated with aquaculture (production of freshwater fish, shrimp, crab or shellfish), human medical care and agricultural production factors [87].

On the other hand, this is also related to the physical and chemical properties of TCs (adsorption and transformation), distance from the source, water environment temperature, pH, salt concentration and bioavailability [88]. The degradation of TCs has been demonstrated to be highly influenced by temperature, and a lower water temperature may make the degradation rate slow, which encourages the persistence of antibiotics in the aquatic environment. Doses tend to increase, taking into account the dilution of surface water by rain during the rainy season [89]. Internal uptake predominates in the bioaccumulation of antibiotics by phytoplankton and zooplankton. After phytoplankton ingests antibiotics, phytoplankton-derived organic matter is transferred to biota with higher nutrient levels or deeper water and sediments, where antibiotics are one of the basic methods of occurrence and destination in the aquatic environment. With increasing phytoplankton biomass and temperature, the antibiotic transfer in the planktonic local ecosystem increased, and the yearly average biomagnification values of OTC and TC ranged between 0.18 and 2.25, suggesting that these compounds may move through the planktonic food web and biomagnify [90,91]. The role of TC in the river was investigated, and reductions in bacterial and algal epiphytic biomass and nematode abundance were observed. Furthermore, at the highest doses of TC (10 μg/L and 100 μg/L), bacterial diversity recovered, but algal and nematode abundance did not recover after 28 d of being untouched [92].

#### 3.4.2. Accumulation of TCs in Water Sediment

The fugacity differential between water and surface sediment drives the exchange of surface water and sediment [93]. Water sediment may be a secondary pollution source. Sediment can serve as an important carrier for the accumulation of TCs and as a potential source of TCs in the water environment when the environment changes. For benthic creatures, sediments serve as both a habitat and a food source. The residual content of TCs in water sediment is summarized (Table 4) to understand that TCs in sediments pose a high potential risk to aquatic ecosystems and human health. The residual levels of TCs in lake, river and marine sediments in the Chinese region range from 0–196.7 μg/kg, 0–422 μg/kg and 0–1478.29 μg/kg, respectively. The Chaobai River, Liao River Basin, Hai River and Yellow Sea TC pollution is more serious than other regions, especially OTC.

The degree of adsorption of TCs is related to the content of organic carbon and clay: the more organic carbon present, the higher the amount of antibiotics adsorbed in the sediment. Sand’s limited capacity for adsorption is caused by its low organic matter concentration, and the variation in antibiotic concentrations in sandy sediments can be explained by the variation in dynamics and contamination load [94]. Since TCs exhibit high organic carbon-water partition coefficients in the environment, the observed variations in TC concentrations upstream and downstream in the Haihe River can be attributed to TCs’ great capacity to bind to particles [95]. TCs may be more common in water and suspended particles than in sediment due to the high rate of TC absorption by suspended particles [96,97]. The Haihe River’s high population density results in low levels of medication consumption, wastewater discharge and TC concentrations in the range of nd-387,000 ng/L [98]. TCs readily form complexes with natural organic matter and heavy metals because they contain hydroxyl and dimethylamino nitrogen atoms [99]. According to studies, the pH of water is a key factor in determining how different antibiotic kinds form a partition between sediment and water. Low pH makes it very simple to transfer antibiotics through sediment, whereas pH increases may lead to lessened antibiotic sorption in sediment [96]. With rising salinity, the adsorption of TC and OTC on sediment declines [100]. Seawater’s ion concentrations are affected by salinity changes, which have an impact on TC sorption by altering the interfacial potential and competing for ion-exchangeable sites [97,101]. It is important to consider other processes involving sediment such as ion exchange, cation bridging, surface complexation and hydrogen bonding [102]. Both the release of antibiotics from soil and the movement of pollutants with bottom currents may be to blame for the stark changes in antibiotic concentrations between surface and near-bottom water. The suburbs have lower TC concentrations than urban and rural areas. In suburban locations, OTC exhibits the greatest potential for accumulation, whereas urban areas show the least potential. Although the most severe antibiotic contamination was found in urban regions, the rate of buildup there was far slower than in suburban and rural areas [101]. The increased sorption of antibiotics by microplastics in freshwater systems compared to seawater may increase their bioavailability, accumulation and long-distance transport in the food chain, increasing the risk to the ecosystem [103].

#### 3.4.3. TC Accumulation in Groundwater

The world’s largest supply of freshwater is groundwater, which is made up of 97% available freshwater and 3% mostly surface water [17]. The most typical method used to dispose of municipal solid waste is landfilling. Solid waste, such as unused or expired prescription drugs and plastic items, can release dangerous substances into landfill leachate, which then affects environments [104]. In April (2014), the concentrations of OTC, TC and DC in the leachate of the Shanghai landfill were 425.1 ng/L, 19.0 ng/L and 98.4 ng/L, respectively, and CTC was not found. Meanwhile, in July, the concentrations of DC, OTC and CTC were 541.9 ng/L, 106.8 ng/L and 496.5 ng/L, respectively, and TC was not found [105]. Due to their greater solubility and persistence, TC metabolites are reported to appear in groundwater at a higher rate than their parent compounds [106]. The presence of antibiotics in groundwater has also attracted worldwide attention. The existence of natural soil infiltration layers hinders the downward migration of TCs, which can remove most TCs from rainfall, irrigation and human activities. In the groundwater of the Han River, the content of TCs in spring was higher than that in autumn, and the concentrations of TC, OTC and CTC were 115.2 ng/L, 28.7 ng/L and 58.1 ng/L, respectively [89]. Ma et al. reported that the maximum concentrations of TC, OTC, CTC and DC detected in groundwater in 15 Chinese cities were 48 ng/L, 39 ng/L, 76 ng/L and 39 ng/L, respectively [107]. Cheng et al. reported that the maximum concentrations of TC, OTC and CTC found in shallow groundwater in northern and southwestern China were 184.2, 237.3 and 184.2, respectively [108]. In July (2019), the maximum concentrations of TC, OTC, CTC and DC in the morning lake of Hanjiang Plain were 2.63 ng/L, 3.91 ng/L, 3.71 and 5.73 ng/L, respectively. In January (2020), the maximum concentrations of TC, OTC and DC in the morning lake of Hanjiang Plain were 2.11 ng/L, 3.73 ng/L and 4.35 ng/L, respectively, and CTC was not found [109]. The above data indicate that TCs may infiltrate the aquifer and pollute the groundwater, thereby threatening public health [110].

**Table 4:** Accumulation of TCs in water and sediment.

Area		TC	OTC	CTC	DC	References
Taihu Lake	Water (ng/L)	nd–87.90 (43.20)	nd–72.80 (44.20)	nd–142.50 (67.90)	/	[111]
	Sediment (µg/kg)	nd–112.20 (47.90)	nd–196.7 (52.8)	nd–48.50 (19.00)	/	
Dongting Lake	Water (ng/L) Sediment (µg/kg)	nd–21.51nd–3.76	/nd–1.34	nd–6.5nd–3.01	//	[112]
Honghu Lake	Water (ng/L)	965.7 (300.50)	2199.50 (217.90)	828.90 (301.20)	/	[113]
Poyang Lake	Water (ng/L)	nd–10.80	nd–48.70	nd–18.10	nd–39.70	[114]
Chaohu Lake	Water (ng/L)	17.80	4.90	4.00	42.30	[115]
Baiyangdian Lake	Water (ng/L)	8.07–85.19	4.64–90.30	/	/	[116]
	Sediment (ng/g)	4.78–93.36	4.28–35.40	/	/	
Liao River Basin	Water (ng/L)	nd–28.65	nd–741.85	nd–25.05	/	[13]
	Sediment (µg/kg)	nd–7.97	nd–384.59	nd–12.26	/	
Chaobai River	Water (ng/L)	nd–124 (16.90)	nd–553 (176)	nd–32.4 (1.61)	/	[117]
	Sediment (ng/g)	nd–58.3 (13.30)	nd–77.7 (12.00)	nd–89.8 (10.20)	/	
	Sediment (µg/kg)	nd–7.97	nd–384.59	nd–12.26	/	
Karst River	Water (ng/L)	101.10	54.60	5.20	/	[118]
Yellow River	Water (ng/L)	18.60–28.50	15.80–21.30	/	/	[119,120]
	Sediment (ng/g)	18.00	184	/	/	
Yellow River Delta	Water (ng/L)	3.65–64.89	4.60–83.54	/	/	[97]
	Sediment (ng/g)	3.22–26.78	1.18–11.49	/	/	
Weihe River	Water (ng/L)	2.20–55.50	nd–32.00	nd–21.70	/	[98]
	Sediment (µg/kg)	2.70–48.80	0.80–38.50	0.90–16.40	/	
Hai River	Water (ng/L)	0–4279	0–97,434	0–13,641		[115]
	Sediment (ng/g)	135 (2.00)	422 (2.52)	10.90	7.00	
Yangtze River	Water (ng/L)	0–79.90	0–78.30	0–40.60	/	[24]
Pearl River	Water (ng/L)	/	0–29.30	0–79.40	/	[121]
	Sediment (µg/kg)	72.6	111	/	/	
Hanjiang River	Water (ng/L)	nd–12	nd–5.90	nd–15	nd–17	[122]
	Sediment (ng/g)	nd–3.00	nd–5.60	nd–11	nd–14	
Huangpu River	Water (ng/L)	nd–54.30	nd–219.80	nd–46.70	nd–112.30	[123]
	Sediment (µg/kg)	nd–21.70	0.60–18.60	nd–6.30	nd–21.30	
Bohai Sea	Water (ng/L)	(16)	(93)	/	/	[124]
	Sediment (µg/kg)	/	1.6–48.40	9.10	/	
Yellow Sea	Water (ng/L)	nd–0.90	nd–42.63	/	nd–6.53	[125]
	Sediment (ng/g)	nd–7.43	nd–1478.29	/	nd–0.26	
Guangdong coastal areas	Water (ng/L)	nd–22.40 (3.78)	nd–914.06 (81.54)	nd–102.38 (22.53)	nd–0.77 (0.26)	[126]

Concentration range of each antibiotic in different regions: (minimum–maximum) or (maximum). nd: not detected.

### 3.5. Pollution Status of TCs in Air Bodies

Due to technical limitations, the presence of antibiotics and transport mechanisms in the air is rarely considered. It is generally considered that the air may contain detectable concentrations of antibiotics in pharmaceutical plants, hospitals, laboratories, livestock farms and feed mills. Airborne particulate matter is a complex biologically active particle that further exacerbates health and environmental hazards as it is loaded with fungi, bacteria and viruses as well as many other components of endotoxins, disinfectants, antibiotics (TCs) and antibiotic resistance genes [106,127]. TCs are usually mixed in feed for therapeutic purposes. Typically in powder or pellet form, this feed can produce a noticeable amount of dust when handled. Dried liquid manure particles may be another source of antibiotics. Large levels of the parent medication are expelled due to the low metabolic rate of TCs in pigs, and these chemicals can leave residue in the liquid feces. TCs have recently been shown to be stable and to accumulate in dried liquid manure particles in environmental samples [128]. In filters collected upwind of feed yards, TCs and CTCs were found. TCs may originate from feed yard manure application sites; deposition and reemission are also possibilities. TCs can be found in stoichiometric concentrations in re-emitted particles compared to rapidly degrading drugs [129]. Antibiotics and ARGs present in atmospheric particles can return to the ground through rain or snowfall, facilitating long-range transmission [130,131]. The presence of antibiotics or antimicrobial resistance genes in atmospheric particles can facilitate transmission through rain and snow. TCs detected in airborne forage particles can have serious effects on the honeybee gut microbiome [132].

## 4. Summary and Future Prospects

Generally speaking, the residue of TCs in manure in China is in the order of mg/kg. Affected by factors such as culture scale, quantity and species preference of TCs, sampling time and place, the residual concentration of TCs in manure varies greatly in different production areas and different research reports. The performance and cost of different treatment units of sewage treatment plants in China are different due to the situation. The residual antibiotics in the effluent and excess sludge are usually in the order of ng/L.

TCs are generally detected in the Chinese environment, and their distribution is irregular. There are large differences in the types and concentrations of TCs in Chinese watersheds, mainly due to the large area of China and the different industrial and agricultural conditions in different places, with the highest emission densities concentrated in the economically developed and densely populated areas of Beijing, Tianjin and Hebei, the Yangtze River Delta and the Pearl River Delta. Antibiotics enter the environment through different forms, and more and more TCs are exposed to the ecological environment in China, such as the detected amount, storage and long-distance transport of antibiotics in the environment. The concentration of TCs in most areas is close to or even above the ecological risk trigger value, which indicates a certain potential environmental risk and therefore requires an environmental risk assessment.

The amount of TCs added to feed to encourage animal growth is far above the amount actually demanded. We need to change the concept of antibiotic use, scientifically regulate its use, establish relevant policies and establish antibiotic supervision and discharge standards. The research and development of alternatives needs to be strengthened, and degradation methods (physical, chemical, microbiological) need to be developed for severely polluted areas, especially in the field of biodegradation. These methods can be developed into promising removal techniques for TCs. However, there are still some problems that keep relevant research in the laboratory stage.

The presence of exogenous antibiotics in air, soil and water environments allows TCs to enter organisms. TCs can be stored in vegetables, crops, animals, by-products and aquatic plants and animals. Although TCs can be stored in vegetables, crops, animals, by-products and aquatic animals and plants, it is unclear whether TCs will disappear in vegetables, fruits and food processing. It is recommended to develop tools for evaluating how the presence of TCs affects human health and to model the migration pathways of TCs from pharmaceutical factories to the environment.

At present, the degradation mechanism and influencing factors of TCs are still unclear. Physical, chemical and biological degradation methods are mostly laboratory-based, with varying degradation capacities and products, and subject to environmental factors (pH, temperature, humidity, etc.). Methods for the determination and extraction of tetracycline and related metabolites also need to be further optimized. At present, most studies only examine the extent to which TCs are reduced, but the degradation products are not studied in detail. Therefore, it is necessary to further understand the potential ecological risks of TCs, TC transformation products and tetracycline resistance genes to the environment. In addition, pollutants such as heavy metal ions, microplastics, chemicals, disinfectants and other types of antibiotics present in the environment have a responsive relationship (antagonistic, synergistic and cumulative), effects on beneficial bacteria in the environment (mycorrhizal fungi and rhizobia) and ecological effects (changes in nitrogen conversion, sulfate reduction, nutrient cycling and organic matter degradation).

## Data Availability

No new data were created or analyzed in this study. Data sharing is not applicable to this article.
